# Work accident or accident at work?

**DOI:** 10.47626/1679-4435-2023-1209

**Published:** 2024-11-14

**Authors:** Claudio José dos Santos Júnior, Silvio Nunes da Silva Júnior, Frida Marina Fischer

**Affiliations:** 1 School of Public Health, Universidade de São Paulo, São Paulo, SP, Brazil; 2 Department of Portuguese Language and Teaching Practices, Universidade de Pernambuco, Garanhuns, PE, Brazil

**Keywords:** accidents, occupational, occupational accidents registry, occupational health, surveillance of the workers health, legislation, labor, acidentes de trabalho, notificação de acidentes de trabalho, saúde ocupacional, vigilância em saúde do trabalhador, legislação trabalhista

## Abstract

This article discusses the differences between the terms “acidente do trabalho” (ie work accident) and “acidente de trabalho” (ie accident at work) on the basis of grammatical and semantic considerations. The syntactic-semantic distinctions of these expressions and the sociological implications of these terms are considered as well as their applicability in the national context of labor and social security legislation and the perspective adopted by international organizations.

## Abstract (pt)

Neste artigo, discutem-se as diferenças entre as nomenclaturas “acidente do trabalho” e “acidente de trabalho” com base em considerações gramaticais e semânticas. São tecidas reflexões sobre as distinções sintático-semânticas dessas expressões e as implicações sociológicas desses termos, bem como sua aplicabilidade no contexto nacional em relação à legislação trabalhista e previdenciária e à perspectiva adotada por organismos internacionais.

## INITIAL CONSIDERATIONS

Although medical literature and regulatory instruments do not explicitly distinguish between “acidente do trabalho” (ie work accident) and “acidente de trabalho” (ie accident at work), semantic and grammatical differences between these terms are emphasized. These differences can be explained by considering the principle of the syntactic-semantic value of prepositions, an aspect that has been the focus of attention in specialized literature within the field of national linguistics and grammar.^1,2^

In the field of Occupational Health, as in other areas of knowledge production, terminological variations can lead to changes at both micro and macro levels, depending on the specific context.^1-3^ These syntactic-semantic differences may reflect different perspectives on the relationship between the accident and the work, and it is therefore important to recognize and understand these subtleties for a comprehensive analysis of occupational risks and preventive measures in the workplace.

## WHAT ARE THE DIFFERENCES?

The term “acidente do trabalho” is formed by the contraction “do” (preposition “de” + definite article “o”), indicating a relationship of possession or belonging. In this case, it suggests that the accident is related to work, without specifying the nature of this relationship. On the other hand, the expression “acidente de trabalho” uses the preposition “de” to establish a more direct link between the accident and the work, indicating that the accident is the result of an action or event related to the work environment. Grammatically, the difference between the two expressions lies therefore in the use of the contraction “do” (“de” + “o”) or the preposition “de.”

Semantically, the construction using “do” allows for a broader interpretation, in which the accident can be related to work in various ways, whether as a direct or indirect consequence, or even as an inherent characteristic of certain occupational activities. On the other hand, the construction using “de” establishes a more restricted link, suggesting that the accident is related to a specific action or circumstance within the work context (Table 1).

**Table 1:** Morphological analysis of the terms “acidente do trabalho” and “acidente de trabalho”

Acidente do trabalho	Acidente de trabalho
“Acidente”: noun, common, singular, masculine.	“Acidente”: noun, common, singular, masculine.
“Do”: preposition “de” + definite article “o” (contraction).	“De”: preposition.
“Trabalho”: noun, common, singular, masculine.	“Trabalho”: noun, common, singular, masculine.
In this case, “do” indicates possession or belonging, meaning the accident belongs to or is related to the work.	In this case, “de” indicates a relationship between the accident and the work. The accident is related to the work but does not indicate possession or belonging.

These constructions not only represent grammatical and semantic distinctions, but also have relevant sociological implications. The term “acidente do trabalho” reflects a comprehensive approach that considers the interactions and conditions present in the work environment that can influence the occurrence of accidents. This perspective is in line with the principles of Ergonomic Work Analysis^4^ and the ideals of the third phase of the scientific study of safety and accident analysis.^5^ In this phase, multiple causality is recognized, where the accident is explained by various factors involving the interaction between the individual, the work situation, group factors, technical-organizational factors, the work environment, and institutional, and social relations.^5^

The term “acidente do trabalho” is also consistent with Reason’s systemic approach to accidents, which emphasizes latent conditions as the origin of accidents.^6^ These latent conditions are related to management decisions, organizational culture, investments, management policies, technologies, and materials used in the organization, maintenance practices, and other aspects.^6^

Llory’s ideas also support the notion that an accident should be understood not only as a dormant or latent factor, but also as a factor incubated within the system.^7^ For him, it is important that accident investigations consider and analyze aspects of the history of the organization, both at the level of the individual dimensions of each member and in the interpersonal, horizontal and vertical relationships that have historically developed and been established in work situations.^7^

The term “acidente de trabalho” tends to emphasize the direct responsibility of the work in the occurrence of the accident, highlighting the need for preventive measures and protection in the work environment. Thus, the use of “acidente do trabalho” can be associated with a more structural approach, which considers factors such as work organization, working conditions, and labor legislation to understand the causes of accidents. On the other hand, the term “acidente de trabalho” is associated with a more individualized, reductionist perspective, which focuses on the worker’s responsibility to avoid accidents by complying with safety standards and the correct use of equipment; in this latter case, the event is considered a simple occurrence resulting from a single cause or a few causes, either human or technical in origin.^5^

## WHICH TERM TO USE?

Based on the points raised above and drawing further on studies of prepositions such as those of Coelho^8^ and Perini-Santos,^9^ it is suggested that prepositions are no longer seen as mere words that connect larger terms, as traditional grammar theory would suggest.

Thus, with reference to Castilho as cited in Berto,^10^ the author points out that the differences between prepositions arise when they are used as connectives and depend on the nature of the syntactic objects they link. In other words, prepositions reveal a syntactic-semantic character precisely because they are placed on the basis of an initial observation of these two grammatical categories.

Both the terms “acidente do trabalho” and “acidente de trabalho” have valid semantic and grammatical bases, reflect different emphases and approaches, and are widely used in legal and labor language to refer to work-related accidents. Therefore, there is no right or wrong term.

Despite this consideration, the use of the term “acidente do trabalho” rather than “acidente de trabalho” is recommended in the Brazilian context for several reasons. First, this broader grammatical construction reflects the view of the country’s labor and social security legislation, which considers not only sudden and violent accidents, but also occupational diseases, work-related illnesses, commuting accidents, and a variety of other equivalent conditions.^11,12^

The essence of this concept is also adopted by the Brazilian Ministry of Health, which includes in its definition of an accident both typical cases and commuting accidents, regardless of the employment and social security status of the injured worker.^13^ In addition, the Ministry recognizes a list of work-related injuries that must be reported in the Sistema de Informação de Agravos de Notificação (SINAN).^14^

Second, the term “acidente do trabalho” allows for a broader, more comprehensive analysis of the working conditions that contribute to the occurrence of accidents, including factors such as work organization, ergonomics, exposure to chemical and biological agents, among others. This systemic approach is essential for the implementation of effective prevention policies aimed at reducing not only typical accidents but also chronic diseases and work-related injuries.^15,16^

In addition, the use of the term “acidente do trabalho” is in line with the perspective of the International Labour Organization (ILO) and various international organizations, which recognize the importance of a broad and integrated approach to the prevention and management of occupational risks.^17-19^ This terminology provides a better understanding of the complex interactions between working conditions, workers’ health and the social determinants involved.

Thus, the use of the term “acidente do trabalho” helps to raise awareness of the importance of prevention among both employers and workers. This linguistic construction emphasizes that occupational safety and health is a shared responsibility of all stakeholders, and not just an individual obligation. This contributes to strengthening the dialogue between social actors and promoting the implementation of effective prevention and protection measures in the workplace.

## IN BRIEF…

In brief, the difference between “acidente do trabalho” and “acidente de trabalho” lies in the syntactic-semantic construction used, as well as in the sociological implications that each expression carries.

While “acidente do trabalho” is more comprehensive and allows for a broader interpretation of the relationship between accidents and work, “acidente de trabalho” emphasizes the direct, specific link between the accident and work activities. These differences may reflect different theoretical and practical approaches to understanding and preventing workplace accidents.

Therefore, although some legal instruments and scientific texts do not make an explicit distinction between “acidente do trabalho” and “acidente de trabalho,” and sometimes even mix or use them as synonyms, it is important to emphasize that there is a syntactic and semantic difference between these expressions. These differences reflect different perspectives on the relationship between the accident and work, and it is essential to recognize and understand these nuances for a thorough and comprehensive analysis of occupational risks and preventive measures in the workplace.

Therefore, considering the legal aspects, the broader and integrated approach that should govern the health care of workers, the alignment with international organizations and the promotion of awareness, the use of the term “acidente do trabalho” is recommended in Brazil. This terminology better reflects the complexity and multiple dimensions of occupational safety and health.
